# The Need for Evidence-Based Aesthetic Dermatology Practice

**DOI:** 10.4103/0974-2077.58518

**Published:** 2009

**Authors:** CL Goh

**Affiliations:** *Senior Consultant and Clinical Professor, Department of Dermatology, National Skin Centre and National University of Singapore, Singapore*

## DEFINITION OF AESTHETIC PRACTICE

There is currently no internationally accepted definition of aesthetic practice. The UK Cosmetic Surgery Interspecialty Committee has defined cosmetic surgery as an area of practice involving *"Operations and other procedures that revise or change the appearance, colour, texture, structure, or position of bodily features, which most would consider otherwise to be within the broad range of 'normal' for that person"*.[1]

The American Board of Cosmetic Surgery has defined cosmetic surgery as *"a subspeciality of medicine and surgery that uniquely restricts itself to the enhancement of appearance through surgical and medical techniques. It is specifically concerned with maintaining normal appearance, restoring it, or enhancing it beyond the average level toward some aesthetic ideal"*.

## STATISTICS ON COSMETIC SURGERY

According to statistics from the American Society for Aesthetic Plastic Surgery (ASAPS) in 2005, there were nearly 11.5 million surgical and nonsurgical procedures performed in the United States.[2] Surgical procedures accounted for nearly 19% of the total, with nonsurgical procedures making up 81% of the total. Since 1997, there has been an increase of 444 percent in the total number of cosmetic procedures. Surgical procedures have increased by 119 percent and nonsurgical procedures by 726 percent.

The top five nonsurgical cosmetic procedures in 2005 were:

1. Botox™ injections (3,294,782)
2. Laser hair removal (1,566,909)
3. Hyaluronic acids: Hylaform™, Restylane™ (1,194,222)
4. Microdermabrasion (1,023,931)
5. Chemical peels (556,172)

Women had nearly 10,500,000 cosmetic procedures performed in 2005, accounting for 91.4% of the total. Men had 985,000 procedures, approximately 9% of the total. People between the ages of 35 and 50 years accounted for the majority of procedures (accounting for 5.3 million (47%) procedures. Those between 51 and 64 years accounted for 24%, patients aged between 19 and 34 years accounted for 24%, those 65 years and older accounted for 5%, and those aged 18 years and under accounted for 1.5%.

The most common procedures for those aged 18 years and under were laser hair removal, microdermabrasion, rhinoplasty (nose reshaping), otoplasty (cosmetic ear surgery), and chemical peel. The majority (48%) of the cosmetic procedures were performed in an office facility, 28% in a free-standing surgi-center, and 24% in a hospital.

Americans spent approximately $12.4 billion on cosmetic procedures in 2008.[2]

## THE SOCIAL IMPLICATIONS OF AESTHETIC MEDICINE

Due to the increasing demand for aesthetic procedures, it is not uncommon for patients to encounter a menu of aesthetic supplies and procedures. These range from skin care products, skin rejuvenation (tightening, pore reduction, blemish removal, smoothening and tightening the skin), anti-wrinkle treatment, acne scar treatment, pigment removal, stretch mark removal, neck lifting, hair restoration, hair removal, breast firming/enlargement, skin whitening, cellulite removal, lip enhancement, tattoo removal, broken capillary treatment, square jaw treatment, nonsurgical facelift, fat removal, anti-aging medicine, hormonal therapy to mesotherapy.

Numerous skin rejuvenation treatment programs (topical creams, skin care products), skin rejuvenation procedures (such as filler and cosmetic botulinum toxin injections, lasers, light devices, radiofrequency devices, and surgical procedures) have been introduced by dermatologists and plastic surgeons and subsequently, by diverse medical specialities. Nonmedical practitioners, *e.g.,* the beauticians and spa operators, have jumped onto the bandwagon to provide such services.

Many of these aesthetic treatments claim to rejuvenate the skin but are not supported by good scientific evidence. Services and procedures that are unproven in efficacy by medical practitioners are often provided at significant cost to patients, which is considered by many medical practitioners to be a deviation from the normal practice of modern medicine. Many medical practitioners have perceived such deviations to be a growing problem that needs to be addressed as it undermines the trust in and professionalism of the medical fraternity.

Concerns have been raised regarding safety issues as well as the quality of such services by the medical profession and the public. Some patients even sustain injuries and complications from these procedures.

## THE MANY CAUSES FOR INCREASED DEMAND FOR AESTHETIC PROCEDURES

Several factors can be attributed to the increasing demand for aesthetic dermatology procedures, namely, (1) the secular consumer culture among the population at large to prolong youthfulness and self-image, (2) economic abundance, in particular, among well paid executives during good economic climates and the large proportion of retiring baby boomers who have accumulated sufficient savings as they reach their retirement age, (3) technological and medical advances whereby new cosmeceutics and devices have been invented to treat cosmetic disorders with minimal downtime and complications, (4) media-driven demand and hype promoted by some beauticians, medical practitioners, and the cosmeceutical and medical devices industries, (5) high-pressure advertising by various media, (6), a breakdown of institutions and cultural constraints, philosophy, policies etc and, (7) a lack of regulatory control that would help to differentiate evidence from nonevidence-based aesthetic procedures and appropriate training and accreditation regulations. All these factors lead to consumer-driven medicine.

Consumer-driven medicine is often industry-driven medicine with a strong motivation for profit which, in turn, leads to questionable practices and infringement of medical ethics. It is often media-driven medicine because the media benefits from consumption by serving as the vehicle of advertising. Advertising encourages needless consumption that may lead to unethical promotions.

## EFFECTS OF INCREASED DEMAND FOR AESTHETIC PROCEDURES

Increased demand for aesthetic procedures has resulted in an ever-increasing number of beauty parlour and charlatans providing scientifically unproven aesthetic services and making unproven claims. There is also an increased interest among medical/dental practitioners offering aesthetic procedures for which they were never trained during their medical training, and a confused public who will not be able to distinguish a medically trained aesthetic physician from one who is not.

This confused public is beginning to lose confidence and trust in the medical profession because some practitioners are offering nonevidence-based aesthetic treatments and merge their treatments with those offered by beauticians and participate with media to promote their treatments, thereby trivializing and commercializing medical services. These commercialized services tend to falsely promote such procedures as having low/no risk, omitting side effects, exaggerating benefits, exaggerating the indications rather than than the limitations of the treatments, overstating good results and hiding complications and poor responses, and tending to make unsubstantiated claims of superiority. This has been referred to as the invisible hand of the marketing department.

## UPHOLDING THE INTEGRITY AND RESPONSIBILITIES OF THE MEDICAL PROFESSION IN AESTHETIC MEDICINE

The medical fraternity has been traditionally regarded as a credible and trustworthy profession that has typically abided by the time - honored Hippocratic Oath. The advice and services offered by medical professionals are often taken seriously with the belief that doctors will always provide scientifically proven and effective procedures. Their medical training and government regulations in most countries often ensure that the profession maintains a high standard of practice.

The public generally trusts medical practitioners to carry out aesthetic procedures rather than to leave it to an untrained beautician. Thus, medical practitioners are attracted by the fairly lucrative and easy way to make a living than to practice conventional medicine. These factors have inevitably driven medical practitioners of diverse specialities into providing aesthetic services. We know that aesthetic medicine, as it is promoted today, generally does not have strong evidence-based rigor in many of the procedures offered. There is a large gap between evidence and practice. There is a tendency for practitioners of aesthetic medicine to move away from science to quackery. This has led to loss of objectivity and of the conventional professional pursuit of excellence which is expected in the practice of medicine.

The problem is further compounded by the fact that, at the moment, there are no hard-and-fast rules governing the way aesthetic procedures like botulinum toxin injections are carried out or promoted. Patient safety is left to the discretion of individual doctors.

In many countries, there is no proper accreditation process to regulate the practice of aesthetic medicine. Many aesthetic practitioners are not adequately trained to carry out aesthetic procedures. This has resulted in patients complaining against medical practitioners. The practice of aesthetic medicine should not be exempted from the need for structured training and accreditation. This ultimately serves to protect the public from unproven and unsafe treatments. Leaving the aesthetic medicine industry to regulate itself is not a feasible solution as professional and ethical standards in an unregulated industry might take a hit in this lucrative business.[4]

In Singapore, the Singapore Medical Council (SMC) Ethical Code and Ethical Guidelines require doctors to treat patients according to generally accepted methods. A doctor shall not offer to patients, management plans or remedies that are not generally accepted by the profession, except in the context of a formal and approved clinical trial. The guiding principles in any medical treatment must be that it is effective and that due cognizance will be given to patient safety. In the context of aesthetic practice, it must go beyond the "Do No Harm" principle and must be seen to benefit the patient positively.

There are many examples to quote to confirm that this principle is often not practiced in aesthetic medicine. For example, one report on the treatment of cellulite with noninvasive devices including massage, radiofrequency, mesotherapy, carboxy therapy etc concluded that "… *no treatment is completely successful as none are more than mildly and temporarily effective*." However, despite the lack of evidence to support efficacy, treatment options continue to proliferate for treating cellulite.[5]

Another report on mesotherapy stated "…*despite the increasing interest in mesotherapy as an alternative method for body contouring, there are few reports of its safety, efficacy, and mechanism of action*." Their study on the efficacy of mesotherapy for body contouring concluded that mesotherapy is not an effective alternative treatment modality for body contouring.[6]

## NEED FOR ETHICAL GUIDELINES FOR THE PRACTICE OF AESTHETIC MEDICINE FOR THE MEDICAL PROFESSION

### Evidence-based medicine/practice

There is a need to implement guiding principles on the practice of aesthetic medicine by the medical profession. Evidence-based practice is probably the best approach. As per the SMC Ethical Code and Ethical Guidelines, doctors are responsible for ensuring that they are competent and adequately trained before performing any treatment or procedure on a patient. He or she should keep abreast of medical knowledge relevant to practice and ensure that clinical and technical skills are maintained. The guiding principles in any medical treatment must be that it is effective and there is due cognizance given to patient safety.

## WHAT IS EVIDENCE-BASED MEDICINE?

Evidence-based medicine (EBM) is defined as *"the conscientious, explicit and judicious use of current best evidence about the care of individual patients"*.[7]

The keywords in the definition are *"conscientious"* which signifies an *active* process which requires learning, practice, and reflection; *"explicit"* which describes it as a *transparent process* used to practice EBM; *"current"* reflecting being *up to date,* and *"best"* which signifies that one should seek the most reliable evidence source to inform practice.

EBM is a way of thinking and working with the sole objective of ensuring improved health of our patients. The term, "evidence-based practice" is often used instead of EBM and is defined as integrating one's clinical expertise with the best external evidence from systematic research.

It should be noted that "therapeutic guidelines" are not the same as EBM. Many dermatology guidelines now incorporate a grading system that describes the quality of evidence used to make recommendations and describe their strength. Searching for relevant information for your patients frequently opens up *more* rather than fewer treatment options. It is estimated that to keep up with the best evidence available; a general physician would have to examine many journal articles daily and throughout his/her life. The trick is to know how to find information efficiently, appraise it critically, and use it well. The techniques and skills needed to find, critically appraise, and use the best evidence available for the care of individual patients, have been developed over two centuries. These techniques and skills are currently best known as EBM.

### Five steps of practicing evidence-based dermatology

1. Asking an answerable structured question generated from a patient encounter.
2. Searching for valid external evidence.
3. Critically appraising the evidence for relevance and validity based on heirarchy of strength in descending order, namely,

- Systematic reviews and meta-analyses of randomized controlled trials
- Randomized controlled trials
- Nonrandomized intervention studies
- Observational studies
- Nonexperimental studies
- Expert opinion

4.Applying the results of that appraisal of evidence back to the patient.

5.Recording the information for the future.

Two filters need to be applied if one is to keep practicing EBM: The first is to discard irrelevant information, and the second is to spend more time looking at a few high-quality papers, as per the concept of hierarchy of evidence.

Suzanne Fletcher and Dave Sackett described "levels of evidence" for ranking the validity of evidence about the value of preventive maneuvers, and then assigned them as "grades of recommendations".[8]

Modifications of the above system have been proposed over the last few years. But basically, all utilize levels of evidence and grades of recommendation (www.cebm.net/levels_of_evidence.asp). Levels of evidence are based on study design and the methodological quality of individual studies. Grades of recommendation are based on the strength of supporting evidence, taking into account its overall level and the considered judgment of the guideline developers.[9,10] Some of the recommendations are enlisted below:

## HARBOUR AND MILLER'S GRADING RECOMMENDATIONS IN EVIDENCE-BASED GUIDELINES

Recommendations are based on levels of evidence which are as follows:[11]

### Levels of evidence

1++: High-quality meta-analyses, systematic reviews of RCTs, or RCTs with a very low risk of bias.
1+: Well-conducted meta-analyses, systematic reviews of RCTs, or RCTs with a low risk of bias.
1−: Meta-analyses, systematic reviews or RCTs, or RCTs with a high risk of bias
2++: High-quality systematic reviews of case-control or cohort studies or high-quality case-control or cohort studies with a very low risk of confounding bias or chance and a high probability that the relationship is causal
2+: Well conducted case-control or cohort studies with a low risk of confounding bias or chance and a moderate probability that the relationship is causal
2−: Case-control or cohort studies with a high risk of confounding bias or chance and a significant risk that the relationship is not causal
3: Non-analytic studies, *e.g.,* case reports, case series
4: Expert opinion

### Grades of recommendations

- At least one meta-analysis, systematic review, or RCT rated as 1++ and directly applicable to the target population, or a systematic review of RCTs or a body of evidence consisting principally of studies rated as 1+ directly applicable to the target population and demonstrating overall consistency of results
- A body of evidence including studies rated as 2++ directly applicable to the target population and demonstrating overall consistency of results, or extrapolated evidence from studies rated as 1++ or 1+
- A body of evidence including studies rated as 2+ directly applicable to the target population and demonstrating overall consistency of results, or extrapolated evidence from studies rated as 2++
- Evidence level 3 or 4 or extrapolated evidence from studies rated as 2+.

## GRADE WORKING GROUP GRADING SYSTEM

Another useful grading system which can be applied to the practice of aesthetic medicine is one proposed by the GRADE working group.[12,13] The following is a useful grading for quality of evidence:

### High

Further research is very unlikely to change our confidence in the estimate of effect.

Several high-quality studies with consistent results

In special cases: One large, high-quality multi-center trial

### Moderate

Further research is likely to have an important impact on our confidence in the estimate of effect and may change the estimate.

One high-quality study

Several studies with some limitations

### Low

Further research is very likely to have an important impact on our confidence in the estimate of effect and is likely to change the estimate.

One or more studies with severe limitations

### Very low

Any estimate of effect is very uncertain.

Expert opinion

No direct research evidence

One or more studies with very severe limitations

## THE COCHRANE SKIN GROUP (www.csg.cochrane.org/en/index.html)

The Cochrane Collaboration (www.cochrane.org), an international voluntary group of reviewers and researchers from a range of professional backgrounds dedicated to producing systematic reviews, was established in 1992. In December 1997, a Cochrane Skin Group was registered with the Cochrane Collaboration to prepare, maintain, and disseminate reviews on the effects of health care for people with dermatological conditions.

The Group has to produce the best evidence about the effects (good or harm) of health care interventions for dermatological diseases. The scope of the Group includes any dermatological problem that leads a person to seek help from a health care practitioner.[3] The Group seeks to find and analyze all evidence on the effectiveness of preventive, medical, and surgical interventions and of different models of health care provision and management of dermatological diseases. This includes evidence about dermatological treatments that are sold over-the-counter or are widely available.

The Cochrane review is systematic, structured, and painstakingly assembled; it minimizes bias and ensures quality. Whenever possible, Cochrane reviews are based only on RCTs because of the major biases associated with other study designs for assessing treatment effects. After approval according to the internal and external refereeing procedures of the Group, the review is published in the *Cochrane Library* (www.cochranelibrary.com).

A useful source for evidence-based dermatology reports can be found in the Cochrane Skin Group review wherein specific reports on treatment and other studies are collated and analyzed based on the strengths of the reports. Examples of such Cochrane reports are:

Laser and photoepilation for unwanted hair growth reported by M. Haedersdal and P.C. Gøtzsche. The authors' conclusion was, *"some treatments lead to temporary short-term hair removal. High quality research is needed on the effect of laser and photoepilation"*.

Laser resurfacing for facial acne scars reported by Jordan R.E., Cummins C.L., Burls A.J.E., Seukeran D.C. The authors' conclusions were, *"the lack of good-quality evidence does not enable any conclusions to be drawn about the effectiveness of lasers for treating atrophic or ice-pick acne scars. Well designed, randomized, controlled comparisons of carbon dioxide versus Erbium:YAG laser are urgently needed."*

## WHAT ARE WE TO DO WHEN THE IRRESISTIBLE FORCE OF THE NEED TO OFFER CLINICAL ADVICE MEETS WITH THE IMMOVABLE OBJECT OF FLAWED EVIDENCE?

All we can do is our best: give the advice, but alert the advisees to the flaws in the evidence on which it is based.

## APPLICATION OF EVIDENCE-BASED PRACTICE IN AESTHETIC MEDICINE IN SINGAPORE

On 1 November 2008, the SMC introduced its guidelines on the practice of aesthetic procedures for Singapore medical practitioners. Aesthetic procedures were classified based on currently available scientific evidence and administratively into List A and List B.

List A: where there is moderate to high level of evidence and/or with local medical expert consensus that the procedure is well-established and acceptable

List B: where there is low or very low level of evidence and/or with local medical expert consensus that the procedure is neither well-established nor acceptable

### Accreditation to perform aesthetic procedures in List A and List B

Medical practitioners who wish to perform aesthetic procedures in List A should submit a List A notification form (together with copies of certificates of training) to the SMC's Aesthetic Procedure Oversight Committee (APOC) for verification as to whether it could be considered a Certificate of Competence (COC).

Aesthetic procedures under List B are currently regarded as having low or very low level of evidence and are not considered as being well-established. Medical practitioners who wish to perform List B aesthetic procedures should list themselves with the SMC's APOC using a prescribed List B notification form before carrying out any List B aesthetic procedure. Doctors may be subject to audit and should comply with requirements set by the SMC's APOC and the Ministry of Health. Proper documentation of the indications and outcomes of the treatments and procedures are of utmost importance.

Medical practitioners who wish to perform procedures that fall within the definition of Aesthetic Practice in the guidelines but which are not found in either List A or List B, will also have to list themselves with the SMC's APOC. The APOC may then decide on the classification of the procedure or further dictate how the doctor should proceed. It is recommended that medical practitioners should not practice such procedures until they have been classified by the SMC's APOC.

### List A aesthetic procedures

This list reflects the aesthetic treatments and procedures that are supported by moderate to high level of scientific evidence and/or have local medical expert consensus that the procedures are well-established and acceptable. They are grouped into noninvasive, minimally invasive, and invasive [Table 1].

**Table 1:** Classification of List A aesthetic procedures

Classification of list A aesthetic procedures	Aesthetic procedures
Non-invasive	Chemical peels
	Lasers (medical)
	Intense pulsed light
	Radiofrequency, infrared and other devices, *e.g.,* for skin tightening procedures
	Photodynamic/Photopneumatic therapy External lipolysis (heat/ultrasound)
Minimally invasive	Botulinum toxin injection
	Filler injection
	Phlebectomy
	Sclerotherapy
	Thread lifts
	Lasers (vascular lesions, skin pigmentation, and skin rejuvenation)
Invasive (to be performed only by doctors who have the appropriate surgical training)	Abdominoplasty
	Blepharoplasty (including double eyelid)
	Breast enhancement or reduction
	Brow lift
	Free fat grafting
	Hair transplantation
	Implants (excluding breast implants)
	Lasers (skin resurfacing)
	Liposuction
	Rhinoplasty
	Rhytidectomy (facelift)
	Dermabrasion (mechanical)

### List B aesthetic procedures

List B contains aesthetic treatments and procedures that are currently regarded as having low/very low level of evidence and/or being neither well established nor acceptable currently. These are:

- Mesotherapy
- Carboxytherapy
- Microneedling dermaroller
- Skin whitening injections
- Stem cell activator protein for skin rejuvenation
- Negative pressure procedures (*e.g.,* Vacustyler™)
- Mechanized massage (*e.g.,* Slidestyler™, endermologie for cellulite treatment)

There will be circumstances in which doctors may wish to practise such low-evidence procedures on patients. In general, these circumstances are:

- All other conventional and sound evidence-based treatments/procedures have been attempted on the patient and have not been shown to produce the desired outcomes
- The procedure has, based on the available evidence, not been shown to carry any risk of significant adverse effects or harm to any patient
- The patient is aware that the procedure is low-evidence in nature and only offered in view of the lack of efficacy of conventional and sound evidence-based treatments and gives specific consent to this on a consent form.

Having satisfied all the above circumstances and documentation, it is still required of doctors to practise List B aesthetic procedures only under highly monitored conditions that enable the efficacy, or lack thereof, of such procedures to be objectively demonstrated. The objectives, methodology, analysis, and findings obtained through such treatments must be of sufficient scientific validity to establish efficacy or otherwise. In addition, patient response should be documented and retained alongside all case records of such treatments. In the event that the procedure yields adverse or neutral outcomes, the practice of the procedure(s) must be terminated.

The patients must not be charged highly profitable fees for such procedures of low evidence, but a fair fee representing the cost of the procedures plus the cost of providing and administering them. Financial documents relating to these procedures must also be retained for the purpose of audit when required. No medical practitioner shall advertise that he or she is performing aesthetic procedures in List B.

## COMPLIANCE WITH SINGAPORE MEDICAL COUNCIL AESTHETIC PROCEDURE GUIDELINES

Any medical practitioner who performs any aesthetic procedure that is not in accordance with these guidelines or with any requirements set by the SMC or MOH will be deemed by the medical profession as being unethical and bringing disrepute to the profession. Such a doctor may be liable for disciplinary action by the SMC.
